# Assessing the Risk of Antibiotic Resistance in Childhood Pneumonia: A Hospital-Based Study in Bangladesh

**DOI:** 10.3390/healthcare13030207

**Published:** 2025-01-21

**Authors:** Sojib Bin Zaman, Naznin Hossain, Md. Taqbir Us Samad Talha, Kashfia Hasan, Rafid Bin Zaman, Raihan Khan

**Affiliations:** 1 Department of Health Sciences, James Madison University, Harrisonburg, VA 22807, USA; khanrk@jmu.edu; 2 Department of Pharmacy, Dhaka Medical College, Dhaka 1000, Bangladesh; naznin241@gmail.com; 3 Friendship NGO, Dhaka 1212, Bangladesh; taqbirtalha@gmail.com; 4 Department of Public Health and Informatics, Jahangirnagar University, Savar 1342, Bangladesh; hprianka41@gmail.com; 5 Department of Civil and Environmental Engineering, Islamic University of Technology, Gazipur 1704, Bangladesh; rafidbinzaman@gmail.com

**Keywords:** prescription drugs, antibiotic resistance, childhood pneumonia, symptomatic management of pneumonia, public health, low- and middle-income countries

## Abstract

**Background:** Approximately two to three children die from pneumonia every hour, and pneumonia is the leading cause of hospitalization for children under five in Bangladesh. Bangladesh has adopted the Pocket Book guidelines by the World Health Organization (WHO) for hospital management of childhood pneumonia. These guidelines recommend the proper use of injectable antibiotic administration. **Objectives:** We assessed and compared the prescription drugs for treating childhood pneumonia following WHO guidelines in a secondary and tertiary hospital in Bangladesh. **Methods:** We conducted a cross-sectional comparative study among children under five years who were admitted to a tertiary hospital, Dhaka Medical College Hospital (DMCH), and a secondary-level hospital, Kushtia District Hospital (KDH), with pneumonia between May 2021 and May 2022. A structured questionnaire was administered to the eligible participants. Additionally, we reviewed the hospital records related to the patient’s treatment. SPSS (Version 28) was used to conduct statistical analysis. **Results:** 316 children were enrolled during the study period, of whom 66.4% were collected from DMCH. There were 65.8% and 24.6% of patients who were classified with severe pneumonia and very severe pneumonia, respectively. In DMCH, the severity of pneumonia percentage was 57.6%, while in KDH, the percentage was 82%. A significant difference was found between the two facilities in diagnosing complicated pneumonia, prescribing the appropriate antibiotics, and ensuring oxygen availability. Amoxicillin was prescribed to 83.5% of the participants, and ceftriaxone was used at a high rate (64.5–70.9%). Combining injections of ceftriaxone with oral amoxicillin or other combinations of antibiotics, both facilities used high frequencies of non-antibiotic corticosteroids. **Conclusions:** Antibiotics were overprescribed, and injections were prescribed at higher levels than WHO recommended. This could pose a threat to antibiotic resistance. There is a need to enforce standard prescribing policies and treatment guidelines to reduce morbidity and mortality among hospitalized children with pneumonia.

## 1. Introduction

Pneumonia remains a critical public health challenge worldwide, particularly affecting children under the age of five. In 2019, pneumonia caused the deaths of 2.5 million people, including 672,000 children, which translates to one child under 5 dying every 47 s [1]. Mortality is relatively higher in low- and middle-income countries (LMICs) with insufficient health coverage, and the quality of care is sub-optimum [2]. Despite significant progress in mortality trends of under-5 children globally, more than 90% of pneumonia-related deaths in children under five globally occur in settings with insufficient resources [3]. In Bangladesh, pneumonia is not only the foremost cause of hospitalization among children under five but also a significant contributor to child mortality rates. In 2018, pneumonia caused the deaths of over 12,000 children under five in Bangladesh, accounting for 13 percent of all child fatalities, which translates to more than one child every hour [4]. The burden of this disease underscores the urgent need for effective management strategies to reduce mortality and morbidity associated with childhood pneumonia.

Recognizing the severity of the issue, Bangladesh has adopted the World Health Organization’s (WHO) *Pocket Book of Hospital Care for Children: Guidelines for the Management of Common Childhood Illnesses* [5]. These guidelines emphasize the appropriate use of antibiotics, particularly injectable antibiotics, to ensure effective treatment outcomes [5]. The proper administration of antibiotics is crucial not only for the timely recovery of affected children but also for preventing the emergence of antibiotic-resistant strains of pathogens, which pose a significant threat to global health.

Despite the adoption of standardized guidelines, there is limited information on how these recommendations are implemented across different levels of healthcare facilities in Bangladesh. Secondary and tertiary hospitals in Bangladesh serve distinct roles within the healthcare system, with tertiary hospitals typically handling more complex cases and possessing greater resources compared to secondary hospitals [6]. This disparity may lead to variations in antibiotic prescription patterns, which can impact the effectiveness of pneumonia management and contribute to broader issues such as antibiotic resistance [7,8].

According to the WHO *Pocket Book of Hospital Care for Children: Guidelines for the Management of Common Childhood Illnesses* [5], the first-line treatment for severe pneumonia is parenteral ampicillin or benzylpenicillin with gentamicin, whereas mild pneumonia can be managed with oral amoxicillin. Physicians can include injectable gentamicin if there is an indication. If the treatment response to the first-line antibiotic is not good, the physician should change the drug and shift to second-line treatment. Injectable ceftriaxone should be used as a second-line treatment for children with severe pneumonia [9]. Pneumonia was defined as having symptoms such as fast breathing and chest indrawing, which need to be treated with oral amoxicillin at home as first-line treatment. Severe pneumonia was defined as any general danger sign with or without fast breathing that necessitates hospitalization for injectable therapy (ampicillin plus gentamicin as first-line treatment and ceftriaxone as second-line treatment if first-line treatment fails) [9].

Despite earlier research on antibiotic treatments in instances of pneumonia among under-5 children [10], no study has been conducted so far that has investigated the variations in pneumonia treatment practices in primary- and tertiary-level public health facilities. This study aims to explore and compare antibiotic prescription patterns for treating childhood pneumonia between secondary and tertiary hospitals in Bangladesh. By analyzing prescription drug practices, the research seeks to identify adherence levels to the WHO Pocket Book guidelines and uncover any discrepancies that may exist between different types of healthcare facilities. Understanding these patterns is essential for several reasons [11]. Firstly, it can highlight areas where guideline adherence is strong or lacking, informing targeted interventions to improve clinical practices on pneumonia. Secondly, it can provide insights into the factors influencing prescription behaviors, such as the availability of medications, healthcare provider training, and institutional protocols. Lastly, it contributes to the broader effort of optimizing antibiotic use, thereby mitigating the risk of antibiotic resistance development. Therefore, we assessed and compared the prescription drugs for treating childhood pneumonia following WHO guidelines in a secondary and tertiary hospital in Bangladesh.

## 2. Materials and Methods

### 2.1. Study Design and Study Timeline

This cross-sectional comparative study was conducted over a seven-month period from May 2022 to May 2023. By employing a cross-sectional design, the study aimed to provide a snapshot of the current management practices within these distinct healthcare settings, facilitating the identification of adherence levels to established guidelines and highlighting areas for improvement.

### 2.2. Study Participants

The study population comprised all children under the age of five who were admitted with a diagnosis of pneumonia to two selected healthcare facilities in Bangladesh: Dhaka Medical College Hospital (DMCH), a tertiary-level institution in Dhaka, and Kushtia District Hospital (KDH), a secondary-level hospital in Kushtia. In addition to providing a greater range of facilities than secondary hospitals, tertiary hospitals also benefit from highly experienced doctors and nurses, contributing to improved health.

A total of 316 samples were collected from these hospitals. Data collection was focused specifically on patients admitted to the pediatric and neonatal departments, as these units are directly involved in the management and treatment of childhood pneumonia.

### 2.3. Eligibility Criteria

Participants were included in the study if they met the following criteria:

- Age: Under five years.
- Diagnosis: Clinically diagnosed with pneumonia based on WHO Pocket Book guidelines.
- Admission: Admitted to the pediatric or neonatal departments of DMCH or KDH during the study period.

Children are classified as having mild, severe, or very severe/complicated pneumonia according to the Integrated Management of Childhood Illness (IMCI) guidelines. However, children diagnosed with bronchiolitis or asthma in the inpatient pediatric department were excluded to maintain focus on pneumonia-specific cases.

### 2.4. Data Collection

A semistructured questionnaire was employed alongside face-to-face interviews conducted with the parents or guardians of hospitalized children diagnosed with pneumonia. Additionally, a thorough review of patient admission files, including prescriptions and treatment sheets, was performed to assess case management practices. These data were collected from three pediatric units/centers in the tertiary hospital and two pediatric units located in the secondary hospital. During the interviews, comprehensive information was gathered, encompassing the child’s age, sex, weight, vital signs, and clinical symptoms. Clinical data collected included the dosage and formulation of prescribed drugs, types of antibiotics used, names and types of bronchodilators, oxygen therapy status, chief complaints documented by physicians, types of pneumonia diagnosed, and investigations prescribed.

The physicians in this study prescribed antibiotics using clinical guidelines and diagnostic tools in accordance with clinical procedures. The diagnosis of pneumonia was determined by assessing the child’s vital signs, including respiratory rate, oxygen saturation, and temperature, as well as by performing a physical examination to evaluate abnormal lung sounds, chest tightening, or respiratory distress.

According to the WHO *Pocket Book of Hospital Care for Children: Guidelines for the Management of Common Childhood Illnesses*, physicians managed pediatric pneumonia and other conditions [5]. This resource offers evidence-based guidelines for assessing pneumonia severity and selecting appropriate first-line antibiotics based on common pathogens and local resistance patterns. Using these guidelines, physicians aim to minimize the risk of antibiotic resistance by targeting the most likely pathogens. Injectable penicillin is recommended for severe pneumonia, whereas oral amoxicillin is recommended for non-severe pneumonia. The structured approach ensures the timely and effective treatment of pneumonia in children. The initial treatment for severe pneumonia cases, such as very severe pneumonia, was guided by one of three cultures: Blood Culture, Nasopharyngeal Swab, or Sputum Culture.

Prescriptions were meticulously reviewed using the authentic drug list to identify the generic names and pharmacological classes of each medication prescribed. We did not include details on the antibiotic doses to keep the report simple and focused. However, we followed the recommended doses outlined in the WHO *Pocket Book of Hospital Care for Children: Guidelines for the Management of Common Childhood Illnesses* [5]. This ensured the accurate classification and analysis of antibiotic usage patterns. Following data collection, all information was rigorously checked for accuracy and completeness. In instances of missing data from the questionnaires, corrective measures such as follow-up interviews or additional record reviews were undertaken.

### 2.5. Statistical Analysis

Data entry was performed using Microsoft Excel to organize the collected information efficiently. Subsequently, statistical analysis was conducted utilizing SPSS software (Version 19) to ensure robust and accurate results. Descriptive statistics were employed to summarize the data, including calculation of proportions, means, and standard deviations. These measures provided a clear overview of the demographic and clinical characteristics of the study population, and the distribution of antibiotic prescriptions across the two hospital settings.

To explore potential associations between categorical variables, cross-tabulation analyses were performed. This approach allowed for the examination of relationships between variables such as hospital type (secondary vs. tertiary) and specific antibiotic usage patterns. The chi-square test was utilized to assess the significance of these associations, determining whether observed differences were likely due to chance or represented meaningful trends within the data. For continuous variables, such as the dosage and duration of antibiotic treatments, unpaired t-tests were conducted to compare means between the two hospital groups. A *p*-value of less than 0.05 was established as the threshold for statistical significance, indicating that the observed results were unlikely to have occurred by random variation alone.

## 3. Results

The study included 316 participants. The mean age of the participants was approximately 3.1 years, with no significant difference between the two hospital groups (*p* = 0.43). The gender distribution was also similar, with males comprising 55.7% of the total sample (Table 1). Regarding the residence of the participants, 61.1% lived in urban areas, with a higher proportion of urban residents in the tertiary hospital group (63.9%) compared to the secondary hospital group (58.2%). The diagnosis of pneumonia showed that 64.5% of the participants had severe pneumonia, with a higher percentage in the secondary hospital group (82.0%) compared to the tertiary hospital (57.6%). Conversely, very severe/complicated pneumonia was more prevalent in the tertiary hospital group (31.4%) compared to the secondary hospital (11.3%) (*p* = 0.02).

Antibiotic prescription patterns revealed significant differences between the two hospital groups. Amoxicillin was prescribed to 83.5% of the participants, with a higher prescription rate in the tertiary hospital group (89.2%) compared to the secondary (77.8%) (*p* = 0.02). Ceftriaxone was prescribed to 64.2% of the participants, with a higher rate in the tertiary hospital group (70.9%) compared to the secondary hospital group (57.6%) (*p* = 0.04). The use of oxygen therapy was significantly higher in the tertiary hospital group (62.0%) compared to the secondary hospital (35.4%) (*p* < 0.01).

Data presented in Table 2 highlights the significant differences in the clinical features and management of pneumonia between DMCH and KDH. Chief complaints such as fever, runny nose, and respiratory distress were common in both hospitals. A higher percentage of patients at DMCH received supportive care measures like nebulization (46.6%) and oxygen (50.9%) compared to KDH (39.6% and 29.2%, respectively).

The investigation practices also varied between the two hospitals. For instance, blood tests such as Hb% and DC were more frequently advised at DMCH (33.8% and 30.0%, respectively) compared to KDH (25.4% and 6.6%, respectively). Similarly, chest X-rays were more commonly performed at DMCH (32.8%) than at KDH (16.0%). These differences in clinical features, management practices, and investigation protocols suggest variations in the approach to pneumonia treatment between the two hospitals, which could be influenced by factors such as resource availability, patient demographics, and hospital protocols.

The study compared the adherence to WHO standard treatment guidelines for pneumonia between the two hospitals, DMCH and KDH (Table 3). The findings revealed significant differences in several areas. At DMCH, 99.0% of doctors identified general danger signs to diagnose pneumonia, compared to 91.1% at KDH (*p* = 0.003). Additionally, 75.2% of doctors at DMCH prescribed the correct antibiotics as per WHO guidelines, while only 62.6% did so at KDH (*p* = 0.032). Supportive care practices also varied, with 99.0% of health workers at DMCH ensuring daily maintenance fluids for children, compared to 86.0% at KDH (*p* = 0.001). Furthermore, 94.0% of doctors at DMCH prescribed oxygen to children with respiratory distress, significantly higher than the 71.0% at KDH (*p* = 0.006).

Monitoring practices showed notable differences as well. At DMCH, 94.0% of nurses checked that nasal prongs were not blocked and were correctly placed, compared to 71.0% at KDH (*p* = 0.006). The adherence to WHO guidelines for antibiotic therapy was 75.2% at DMCH and 62.6% at KDH (*p* = 0.032). These results indicate that DMCH generally had higher adherence to WHO guidelines across various aspects of pneumonia treatment, highlighting areas where KDH could improve its practices to align more closely with international standards.

Table 4 highlights the use of antibiotics and non-antibiotics in treating pneumonia patients at DMCH and KDH. The most prescribed single antibiotic at both hospitals was ceftriaxone, with 25.7% of patients at DMCH and 22.6% at KDH receiving it. Combination antibiotic therapies were also prevalent, with ampicillin and gentamicin being the most frequently used combination at KDH (20.8%) compared to DMCH (14.8%). Notably, the use of meropenem was higher at DMCH (2.9%) than at KDH (0.8%), indicating a preference for broader spectrum antibiotics at DMCH.

In terms of non-antibiotic treatments, corticosteroids were the most prescribed at both hospitals, with a slightly higher usage at KDH (63.2%) compared to DMCH (55.7%). Bronchodilators were more frequently prescribed at DMCH (37.1%) than at KDH (28.3%). The use of mucolytics and anti-leukotrienes also varied, with DMCH showing a higher usage of anti-leukotrienes (17.1% vs. 11.3%) and KDH having a higher prescription rate of mucolytics (17.0% vs. 10.0%). These variations in treatment approaches reflect the differing clinical practices at the two hospitals.

Culture sensitivity tests were only conducted for children diagnosed with severe or very severe pneumonia based on their vital signs and physical examination findings. The pathogens identified include *Haemophilus influenzae, Klebsiella pneumoniae, Escherichia coli, Pseudomonas aeruginosa,* and *Staphylococcus aureus,* which are commonly associated with pneumonia (Table 5). The table presents an overview of the bacteria isolated through three culture techniques: Blood Culture, Nasopharyngeal Swab, and Sputum Culture, totaling 286 occurrences conducted in the DMCH (tertiary) and KDH (secondary) hospitals.

The Blood Cultures most frequently detected were *Haemophilus influenzae* (15 counts), *Staphylococcus aureus* (13 counts), and *Klebsiella pneumonia* (10 counts). In contrast, Nasopharyngeal Swabs and Sputum Cultures revealed Streptococcus pneumoniae (19 and 21 counts) as the prevalent isolates. This variation in pathogen distribution underscores the importance of selecting appropriate culture techniques for the accurate diagnosis and effective treatment of pneumonia.

## 4. Discussion

We found that while the treatment of pneumonia closely aligns with the WHO standard treatment guidelines, there are notable differences in the clinical management practices between secondary and tertiary health facilities. The significant differences in antibiotic prescription patterns and the use of oxygen therapy between the two hospitals highlight variations in clinical management practices. The higher prescription rates of amoxicillin and ceftriaxone in the tertiary hospital may indicate a preference for these antibiotics in more severe or complicated cases or differences in clinical guidelines and physician preferences. The significantly higher use of oxygen therapy in tertiary hospitals suggests that these hospitals are better equipped to provide advanced respiratory support, which could be crucial for managing severe pneumonia cases. There is a possibility that the tertiary hospital receives more severe cases, which may explain the higher usage of oxygen therapy, bronchodilators, and broad-spectrum antibiotics, such as meropenem, compared to the secondary-level hospital. This difference is partly due to tertiary hospitals having access to more comprehensive healthcare facilities and resources. This underscores the importance of standardizing treatment protocols and ensuring equitable access to essential medical interventions across different hospital settings.

The study emphasizes the importance of improving healthcare provider training and resource allocation to enhance the management of childhood pneumonia and guide equitable health policy decisions. The higher incidence of pneumonia with complications is a potential gap in early detection and intervention strategies. This gap could be bridged by implementing targeted training programs for healthcare providers, enhancing their ability to identify and manage complications more effectively. Furthermore, findings suggest that secondary-level hospitals could benefit from increased access to diagnostic tools and supportive care to better manage pneumonia cases. Policymakers should consider these insights when designing health policies to ensure the equitable distribution of resources.

The study underscores the importance of optimizing antibiotic use to combat resistance and improve patient outcomes. The higher use of broad-spectrum antibiotics like meropenem could indicate a more aggressive approach to treating severe infections, which may be influenced by local resistance patterns or clinical guidelines [12]. Additionally, the preference for certain antibiotic combinations highlights the importance of understanding local microbial landscapes and resistance patterns to optimize antibiotic stewardship. It should be noted that several factors contribute to antibiotic resistance, including the overuse and misuse of antibiotics, incomplete treatment regimens, and inadequate infection control measures. The spread of resistant bacteria in healthcare settings and the use of antibiotics in agriculture are also important factors to consider. Addressing these factors requires both healthcare practices and public awareness.

According to a study, gentamicin, ampicillin, crystalline penicillin, and cefotaxime were the most prescribed antibiotics in eastern Nepal [13]. Another study [14] conducted in a tertiary hospital in Bangladesh reported that ampicillin, gentamicin, amoxicillin, cloxacillin, and ceftriaxone were widely recommended in hospitals. However, adherence to WHO guidelines was not evaluated in the study. Regardless of the patient’s age group, the most frequently prescribed antibiotic in a tertiary hospital in Malaysia was amoxicillin with clavulanate, followed by erythromycin, cefuroxime, ceftriaxone, and ampicillin. This prescription practice might be influenced by physicians’ personal choices and limited drug-related experience [15]. Similarly to other studies carried out in Gujarat, India, ampicillin, gentamicin, and ceftriaxone were suggested as the most important antimicrobial drugs for respiratory tract infections [16].

Compared to a study conducted in Bangladesh, this study showed a significant use of antibiotics after admission [15]. It also appeared in another study that physicians in primary healthcare in that country have an antibiotic preference for the cephalosporin group [17]. This study highlights significant variations in antibiotic prescribing practices, emphasizing the need for standardized guidelines to ensure the effective use of antibiotics in pediatric care. This study, 14,053 antibiotics were prescribed for 316 patients; this indicated around 4.2 antibiotics per prescription. Over half of the children were treated with a combination of antibiotics, and the rest were treated with a single antibiotic. However, the number of children treated with three antibiotics was small (3–4%). This finding is higher than a study conducted at Jimma University specialized hospital, which showed that the average number of antibiotics per patient was 2.17 [18]. Another study conducted in Hawassa showed that the mean number of antibiotics prescribed per prescription was 1.18 [19]. These differences may be due to variations in prescribing habits among different hospitals or physicians. In addition, the selection of antibiotics in the previous studies may be based on culture sensitivity results. It could be due to better technological advancement and expertise knowledge in both University specialized hospitals than in the selected hospitals of this study.

Hospital practices and guidelines significantly impact the selection of treatment regimens, as they are often tailored to the hospital’s resources, patient demographics, and the severity of the cases managed at the hospital. For example, tertiary hospitals that handle more complex and severe cases may follow protocols emphasizing advanced interventions, such as broad-spectrum antibiotics or specialized therapies. Secondary-level hospitals, on the other hand, may adopt treatment guidelines based on the limited resources available or that focus on less severe conditions. In addition to optimizing patient outcomes, these institutional practices consider the context in which care is provided.

WHO usually recommends 10% of injectable drugs among the total prescriptions for a healthcare setting, and the antibiotic prescription practices in this study are higher than these recommendations [20]. In this study, 92% of patients were treated with injectable antibiotics, and a small portion was treated with oral antibiotics. However, this is practically equal according to a study conducted in Dessie Referral Hospital, which showed that 76% of antibiotics were given through the parenteral route [21]. A retrospective assessment conducted at Mekelle General Hospital showed that the percentage of antibiotic injections in inpatient departments was 95.2% [22]. This variation could be due to differences in sample size, the experience of prescribers and dispensers, and the availability of guidelines to monitor antimicrobial use. In our study, the most used antibiotics and combinations was ceftriaxone followed by an ampicillin + gentamicin (20.4%) combination. This was somewhat similar to the findings of the Jimma University specialized hospital, which showed that the most commonly prescribed combination therapy was ampicillin plus gentamicin (21.9%), followed by ceftriaxone and gentamicin (18.1%) [18].

The differences in non-antibiotic treatments, such as the higher use of corticosteroids, suggest variations in supportive care practices that could impact patient outcomes. The higher prescription rates of bronchodilators may reflect a focus on managing respiratory symptoms more aggressively. These findings highlight the importance of continuous medical education and the implementation of evidence-based guidelines to harmonize treatment practices. The higher adherence to WHO guidelines at DMCH suggests that strict adherence to standardized treatment protocols can lead to better patient care and potentially improved health outcomes. This highlights the need for the continuous training and monitoring of healthcare providers to ensure they are well-versed in and consistently apply these guidelines. The significant differences in adherence rates between the two hospitals also highlight the importance of institutional policies and support systems in facilitating guideline adherence.

### Study Limitations and Strengths

This study has several limitations that should be acknowledged. Firstly, patient care indicators were not explored, which limits the ability to assess the quality of care provided beyond adherence to treatment guidelines. This omission means that important aspects such as patient outcomes, satisfaction, and long-term health impacts were not evaluated, potentially overlooking critical dimensions of healthcare quality. Another limitation is the potential for desirability bias. Participants, particularly healthcare providers, may have altered their behavior or responses to align with what they perceived as desirable or expected by the researchers. This bias could affect the accuracy of the data, leading to an overestimation of adherence to guidelines and the quality of care provided. Furthermore, the results of this study cannot be generalized to other hospitals because the study was conducted only in two medical facilities, one of which was a tertiary institution and one of which was a secondary facility. Since only one institution was selected for each category, it is not possible to make broader generalizations at the institutional level. Therefore, our findings should be interpreted cautiously and viewed as indicative rather than conclusive, emphasizing the need for further research involving a larger and more diverse sample of hospitals to achieve greater generalizability.

Despite these limitations, the study has notable strengths. Firstly, it can highlight areas where guideline adherence is strong or lacking, informing targeted interventions to improve clinical practices. Secondly, it can provide insights into the factors influencing prescription behaviors, such as the availability of medications, healthcare provider training, and institutional protocols. Thirdly, it contributes to the broader effort of optimizing antibiotic use, thereby mitigating the risk of antibiotic resistance development. Finally, the use of high-quality data and the involvement of trained physicians in diagnosing pneumonia ensures the reliability and validity of these findings. These strengths enhance the credibility of the study and provide a solid foundation for future research to build upon.

## 5. Conclusions

The findings indicate that antibiotics are over-prescribed, particularly in injectable forms, exceeding WHO prescribing indicators for treating pneumonia patients. Additionally, the number of drugs per prescription surpasses acceptable levels compared to WHO standards. It is essential to develop and enforce standard prescribing policies and treatment guidelines in Bangladesh to address these issues. Implementing regulatory policies is urgent to promote the rational use of drugs in treating hospitalized children with pneumonia, ultimately preventing child morbidity and mortality.

## Figures and Tables

**Table 1:** Demographic and clinical characteristics of study participants.

Variable	Total (n = 316)	DMCH, Tertiary (n1 = 210)	KDH, Secondary (n2 = 106)	*p*-Value
Age (Mean ± SD), years	3.1 ± 1.4	3.2 ± 1.5	3.1 ± 1.4	0.43
Gender				
Male	176 (55.7%)	113 (53.8%)	61 (57.6%)	0.45
Female	140 (44.3%)	97 (46.2%)	45 (42.4%)	
Residence				
Urban	193 (61.1%)	134 (63.9%)	62 (58.2%)	0.34
Rural	123 (38.9%)	76 (36.1%)	44 (41.8%)	
Pneumonia categories				0.02
Mild	30 (9.5%)	19 (9.0%)	11 (10.3%)	
Severe	204 (64.5%)	121 (57.6%)	83 (82.0%)	
Very severe/complicated	82 (26.0%)	70 (31.4%)	12 (11.3%)	
Antibiotic prescribed				
Amoxicillin	264 (83.5%)	187 (89.2%)	82 (77.8%)	0.02
Ceftriaxone	203 (64.2%)	145 (70.9%)	61 (57.6%)	0.04
others	72 (22.8%)	42 (20.3%)	79 (25.3%)	0.32
Use of oxygen therapy				
Yes	154 (48.7%)	130 (62.0%)	38 (35.4%)	<0.01
No	162 (51.3%)	80 (37.9%)	68 (64.6%)	

DMCH, Dhaka Medical College Hospital; KDH, Kushtia District Hospital; SD, Standard Deviation.

**Table 2:** Clinical features and investigations conducted in the tertiary- and secondary-level hospitals.

Category	DMCH, Tertiary (n1 = 210)	KDH, Secondary (n2 = 106)
Chief Complaints		
Fever	124 (59.0%)	67 (63.2%)
Runny nose	108 (51.4%)	72 (67.9%)
Respiratory distress	76 (36.1%)	43 (40.5%)
Not able to feed	49 (23.3%)	29 (27.3%)
Lethargic	42 (20.0%)	15 (14.1%)
History of Convulsion	21 (10.0%)	10 (29.2%)
Investigations Advised by the Doctor		
Blood	71 (33.8%)	27 (25.4%)
Nasal swab	63 (30.0%)	17 (6.6%)
Culture	26 (12.3%)	5 (4.7%)
Chest X-ray	69 (32.8%)	18 (16.0%)
Supportive Care Provided		
Nebulization given	98 (46.6%)	42 (39.6%)
Oxygen given	107 (50.9%)	31 (29.2%)

DMCH, Dhaka Medical College Hospital; KDH, Kushtia District Hospital.

**Table 3:** Distribution of respondents by adherence to WHO standard treatment.

WHO Standard Treatment Criteria	DMCH, Tertiary (n1 = 210)	KDH, Secondary (n2 = 106)	*p*-Value *
	Number (%)	Number (%)	
Doctors identified the presence of any general danger signs to diagnose pneumonia	206 (99.0)	97 (91.1)	0.003 ^s^
Doctors prescribed the correct antibiotics as per the WHO guideline	157 (75.2)	67 (62.6)	0.032 ^s^
Supportive care			
Doctors prescribed paracetamol if the child had a fever	122 (98.0)	64 (98.1)	0.044 ^s^
Doctors prescribed bronchodilator and/or started steroids if rhonchi were present	60 (96.6)	33 (96.0)	0.837 ^ns^
Health workers (doctor and nurse) ensured that the child received daily maintenance fluids	208 (99.0)	92 (86.0)	0.001 ^s^
Doctors prescribed oxygen to all children with respiratory distress	100 (94.0)	22 (71.0)	0.006 ^s^
Health workers ensured continuous oxygen supply, either as cylinders or oxygen concentrators	106 (99.0)	28 (90.0)	0.01 ^s^
Monitoring			
Nurses checked that the nasal prongs were not blocked and were in the correct place	100 (94.0)	22 (71.0)	0.006 ^s^
Adherence to the WHO standard treatment guideline (corresponds to antibiotic therapy)	157 (75.2)	67 (62.6)	0.032 ^s^

DMCH, Dhaka Medical College Hospital; KDH, Kushtia District Hospital. s = Significant; ns = Not significant; considering *p* < 0.05; * *p* determined by Chi-Square test.

**Table 4:** Use of antibiotics and non-antibiotics in pneumonia patients.

Antibiotics	Formulation	DMCH, Tertiary (n1 = 210)	KDH, Secondary (n2 = 106)
Prescribed single antibiotics for sick children			
Ceftriaxone	Injectable	54 (25.7%)	24 (22.6%)
Ampicillin	Injectable	11 (5.2%)	8 (7.5%)
Gentamicin	Injectable	9 (4.2%)	5 (4.7%)
Amikacin	Injectable	11 (5.2%)	6 (5.7%)
Meropenem	Injectable	6 (2.9%)	1 (0.8%)
Vancomycin	Injectable	3 (1.4%)	2 (1.9%)
Cefixime	Oral	3 (1.4%)	7 (6.3%)
Ceftazidime	Injectable	2 (0.9%)	2 (1.9%)
Prescribed combination of antibiotics for sick children			
Ampicillin + Gentamicin	Injectable	31 (14.8%)	22 (20.8%)
Ceftriaxone + Amikacin	Injectable	18 (8.6%)	9 (8.5%)
Ceftriaxone + Gentamicin	Injectable	3 (1.4%)	2 (1.9%)
Ceftriaxone + Flucloxacillin	Injectable	11 (5.2%)	8 (7.5%)
Ceftazidime + Amikacin	Injectable	9 (4.3%)	7 (6.6%)
Ampicillin + Gentamicin + Ceftriaxone	Injectable	3 (1.4%)	5 (4.7%)
Meropenem + Vancomycin	Injectable	2 (0.9%)	2 (1.9%)
Meropenem + Amikacin + Vancomycin	Injectable	2 (0.9%)	1 (0.8%)
Ceftriaxone + Amoxycillin	Injectable + Oral	6 (2.9%)	9 (8.3%)
Amoxycillin + Gentamicin	Injectable	3 (1.4%)	5 (4.7%)
Amoxycillin + Clavulanic acid	Oral	3 (1.4%)	2 (1.9%)
Prescribed non-antibiotics for sick children			
Corticosteroids	Injection	117 (55.7%)	67 (63.2%)
Bronchodilator	Syrup + Tablet	78 (37.1%)	30 (28.3%)
Mucolytics	Syrup	21 (10.0%)	18 (17.0%)
Anti-leukotriene	Syrup + Tablet	36 (17.1%)	12 (11.3%)
Paracetamol	Syrup + Tablet	63 (30.0%)	38 (35.8%)
Nasal drop	Drop	11 (5.2%)	13 (12.3%)
Multivitamin	Syrup + Tablet	3 (1.4%)	12 (11.3%)
Anti-ulcerate	Syrup + Tablet	4 (1.9%)	15 (14.2%)

DMCH, Dhaka Medical College Hospital; KDH, Kushtia District Hospital.

**Table 5:** Distribution of bacteria isolated across different culture techniques in pneumonia cases (n = 286).

Blood Culture	Nasopharyngeal Swab	Sputum Culture
*Haemophilus influenzae* (15)	*Escherichia coli* (12)	*Escherichia coli* (14)
*Klebsiella pneumoniae* (10)	*Haemophilus influenzae* (18)	*Haemophilus influenzae* (16)
*Escherichia coli* (5)	*Klebsiella pneumoniae* (15)	*Klebsiella pneumoniae* (7)
*Pseudomonas aeruginosa* (16)	*Pseudomonas aeruginosa* (13)	*Pseudomonas aeruginosa* (10)
*Staphylococcus aureus* (13)	*Staphylococcus aureus* (14)	*Staphylococcus aureus* (20)
*Streptococcus pneumoniae* (19)	*Streptococcus pneumoniae* (19)	*Streptococcus pneumoniae* (21)
Others (10)	Others (8)	Others (11)

## Data Availability

Data presented in this study are available on request from the corresponding author.
